# Mitochondria chaperone GRP75 moonlighting as a cell cycle controller to derail endocytosis provides an opportunity for nanomicrosphere intracellular delivery

**DOI:** 10.18632/oncotarget.17234

**Published:** 2017-04-19

**Authors:** Zhihui Gao, Xiuran Niu, Qing Zhang, Hang Chen, Aiai Gao, Shanshan Qi, Rong Xiang, Mattias Belting, Sihe Zhang

**Affiliations:** ^1^ Department of Biochemistry & Cell Biology, School of Medicine, Nankai University, Tianjin, China; ^2^ Department of Clinical Laboratory, Cancer Hospital of Tianjin Medical University, Tianjin, China; ^3^ Department of Clinical Sciences, Section of Oncology, Lund University, Lund, Sweden

**Keywords:** GRP75, cell cycle, clathrin-mediated endocytosis, clathrin-independent endocytosis, nanomicrosphere

## Abstract

Understanding how cancer cells regulate endocytosis during the cell cycle could lead us to capitalize this event pharmacologically. Although certain endocytosis pathways are attenuated during mitosis, the endocytosis shift and regulation during the cell cycle have not been well clarified. The conventional concept of glucose-regulated proteins (GRPs) as protein folding chaperones was updated by discoveries that translocated GRPs assume moonlighting functions that modify the immune response, regulate viral release, and control intracellular trafficking. In this study, GRP75, a mitochondria matrix chaperone, was discovered to be highly expressed in mitotic cancer cells. Using synchronized cell models and the GRP75 gene knockdown and ectopic overexpression strategy, we showed that: (1) clathrin-mediated endocytosis (CME) was inhibited whereas clathrin-independent endocytosis (CIE) was unchanged or even up-regulated in the cell cycle M-phase; (2) GRP75 inhibited CME but promoted CIE in the M-phase, which is largely due to its high expression in cancer cell mitochondria; (3) GRP75 targeting by its small molecular inhibitor MKT-077 enhanced cell cycle G1 phase-privileged CME, which provides an opportunity for intracellular delivery of nanomicrospheres sized from 40 nm to 100 nm. Together, our results revealed that GRP75 moonlights as a cell cycle controller and endocytosis regulator in cancer cells, and thus has potential as a novel interference target for nanoparticle drugs delivery into dormant cancer cells.

## INTRODUCTION

A moonlighting protein is a single protein with multiple functions that are not a result of gene fusions, splice variants, proteolytic fragments, families of homologous proteins, or promiscuous enzyme activities [1, 2]. Several hundred moonlighting proteins have been experimentally identified. Some can simultaneously perform unrelated functions, but others can switch functions due to changes including the cell type, cellular redox status, subcellular location, oligomeric state, and binders of the protein [3–5]. There is evidence that a moonlighting protein can provide a connection between distinct biochemical processes, help the cell to adapt to microenvironmental stress, and contribute to phenotype-corrupted diseases [6–8].

Chaperone proteins are traditionally known as cytoplasmic proteins that prevent clients from misfolding, promote refolding, and correct the assembly of unfolded polypeptides. However, accumulating evidence showed that they have a growing number of moonlighting functions in and/or outside the cell. This is particularly true for the bacterial cell surface-located Cpn60 and DnaK, which generally function as adhesins for a range of host components including plasminogen, glycosphinngolipids, mucins, and CD43 [9–18]. These cell surface-resident Hsp60 and Hsp70 not only control bacterial-host cell interaction, but also stimulate cytokine production, thus exhibiting distinct biological functions [6]. The eukaryotic cell secreted Hsp90 was reported to bind with and stabilize matrix metalloproteinase (MMPs) activation, which contributes to angiogenesis, cancer cell invasiveness, and wound-healing [19–22]. When shuttling into the nucleus, Hsp90 was found to alter MMPs and E-cadherin production, giving rise to a epithelial–mesenchymal transition and enhanced cancer cell motility [23]. Another example of a eukaryotic chaperone is Hsp60 which moonlights as a cell surface receptor for HDL and apoA-II [24]. Interesting, the capacitation of murine sperm requires the presence of cell surface Hsp60 and its tyrosine phosphorylation [25]. All these suggest that the subcellular localization change of cytoplasmic chaperones determines their moonlighting function. However, no organelle-resident chaperone has been investigated.

GRP75 (mortalin/PBP74/mtHSP70), a mitochondrial matrix-resident chaperone protein, is often detected in different compartments of cancer cells. Although GRP75 was originally identified as having a chaperone role in bioenergetics-associated transport and refolding of clients inside mitochondria, it also acts as a guardian against senescence and apoptosis from various stresses, or serves as a safeguard to promote cell proliferation and survival, suggesting its multiple additional functions alter in cancer cells [26, 27]. Notably, GRP75 has recently been characterized as an important cell cycle controller in tumor cells. GRP75 depletion or inhibition causes G0/G1-phase cell cycle accumulation in medullary thyroid carcinoma cells [23, 28]. GRP75 knock down or blockage induces G2/M-phase cell cycle arrest in B-Raf/K-Ras-mutated melanoma and lung cancer cells[29, 30]. Furthermore, the role of GRP75 in G2/M-phase enrichment was also demonstrated in ovarian cancer cells [26]. Previously we accidentally found that GRP75 is enriched as a functional constituent in heparan sulfate proteoglycan (HSPG)-mediated and membrane raft-associated endocytosis vesicles in cervix cancer cells [31]. More recently, we further found that GRP75 promotes clathrin-independent endocytosis (CIE) but inhibits clathrin-mediated endocytosis (CME) by the Cdc42 and RhoA concurrent activation mechanism [32]. Given that endocytosis and the cell cycle are tightly linked and several studies indicated that certain endocytosis pathways are shut down during mitosis [33–38], the endocytosis regulation function of GRP75 may be modulated during the cell division process.

In this study, we investigated whether GRP75 moonlights as a cell cycle control and endocytosis regulator in HeLa and Cos-7 cells. We also determined the effect of GRP75 subcellular localization on its cell cycle control and endocytosis regulation function, and further explored the possibility of potential delivery of different size nanomicrospheres by GRP75 targeting. Our data showed that: (1) CME was inhibited whereas CIE was unchanged or even up-regulated in the M-phase of the cell cycle; (2) GRP75 inhibited CME but promoted CIE in the M-phase, which is due to its high expression in cancer cell mitochondria; and (3) GRP75 targeting enhanced cell cycle G1-phase-privileged CME, which provides an opportunity for intracellular delivery of different size nanomicrospheres. Together, our results reveal that GRP75 moonlighting functions as a cell cycle controller and endocytosis regulator in cancer cell, and thus has potential as a novel interference target for nanoparticle intracellular drug delivery.

## RESULTS

### Reduced CME versus unaltered CIE in the M-phase of the cell cycle

**Figure 1:**
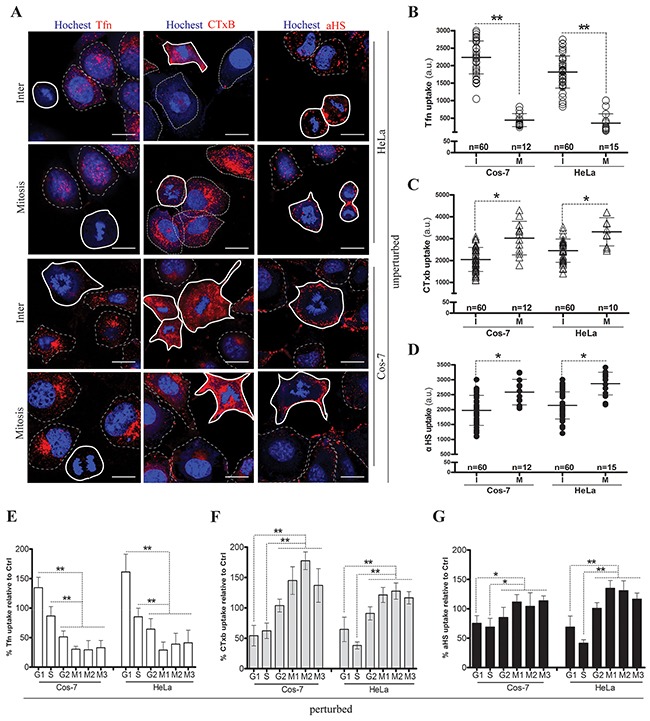
Cell cycle dependent endocytosis variations HeLa and Cos-7 cells were respectively incubated with Tfn-AF647 (25μg/ml, 15min), CTxB-AF647 (10μg/ml, 30min) and αHS-AF647 complex (1:20, 1h) at 37 °C, rinsed with PBS/0.5M NaCl and visualized via confocal microscopy. Representative images from three independent experiments are shown in **(A)**. Scale bar: 20 um. Scatterplots depict the uptake variability of indicated ligands in single cell populations. The uptake quantification for each cell was described in ‘Materials and methods’, and uptake levels in M or I phase cells are summarized in **(B)**, **(C)** and **(D)**. 60 cells were counted for the I phase (polygons with thin dotted line) and ≥10 cells were counted for the M phase (polygons with bold solid line) in each experiment, n=3. Phase-arrested cells were induced by drug treatments as follows: the G1-phase was induced by atorvastatin, the S-phase was induced by double thymidine block, the G2-phase was induced by RO3306 treatment, and the M-phase was enriched by double thymidine block with further nocodazole incubation (M1) or with further 10% FBS stimulation (M2) or with colcomid incubation (M3). Arrested cells were incubated respectively with indicated drugs at 37 °C, and uptakes were immediately analyzed via FACS. The uptake quantification of ligands in any sub-phase was corrected by the corresponding cell cycle phase percentage, and the uptake levels are summarized in **(E)**, **(F)** and **(G)**. 10,000 cells were counted per treatment in each experiment, n=3. Statistically significant differences in relation to the control (no drug treated cells) are shown: **P <0.01, *P <0.05.

It is debated whether endocytosis is totally inactive in mitosis [34, 36, 37]. We first checked endocytosis variation in unperturbed HeLa and Cos-7 cells. Confocal imaging analysis showed that the uptake of the CME marker Tfn was almost shutdown in mitotic cells (Figures 1A, 1B). However, the uptake of the membrane raft marker CTxB was slightly increased in a partial population of mitotic cells (Figures 1A, 1C). In contrast, only a small uptake difference of the αHS complex between mitotic- and interphase-cells was observed (Figures 1A, 1D). In addition, no macropinocytosis was detected unless cells were heavily starved for a long time (Supplementary Figure 5). To clarify this variation, we next synchronized cells in different sub-phases by chemical drug pretreatment (Supplementary Figure 1), and then determined the uptakes in arrested cell cycle phases by flow cytometry measurement. The results showed that, compared with the uptake in atorvastatin-induced G1-phase cells, Tfn uptake was decreased in the double thymidine-blocked S-phase and markedly decreased in the nocodazole/colcomid-induced M-phase (Figure 1E). Conversely, the uptake of CTxB was slightly increased in the Ro3306-induced G2-phase and significantly enhanced in induced M-phase cells (Figure 1F). Only a limited uptake increase of the αHS complex was observed in induced G2/M-phase cells (Figure 1G). These results suggest that CME is decreased whereas CIE remained constant or was even increased in the cell cycle M-phase.

### GRP75 is highly expressed in mitochondria in the G2/M-phase

**Figure 2:**
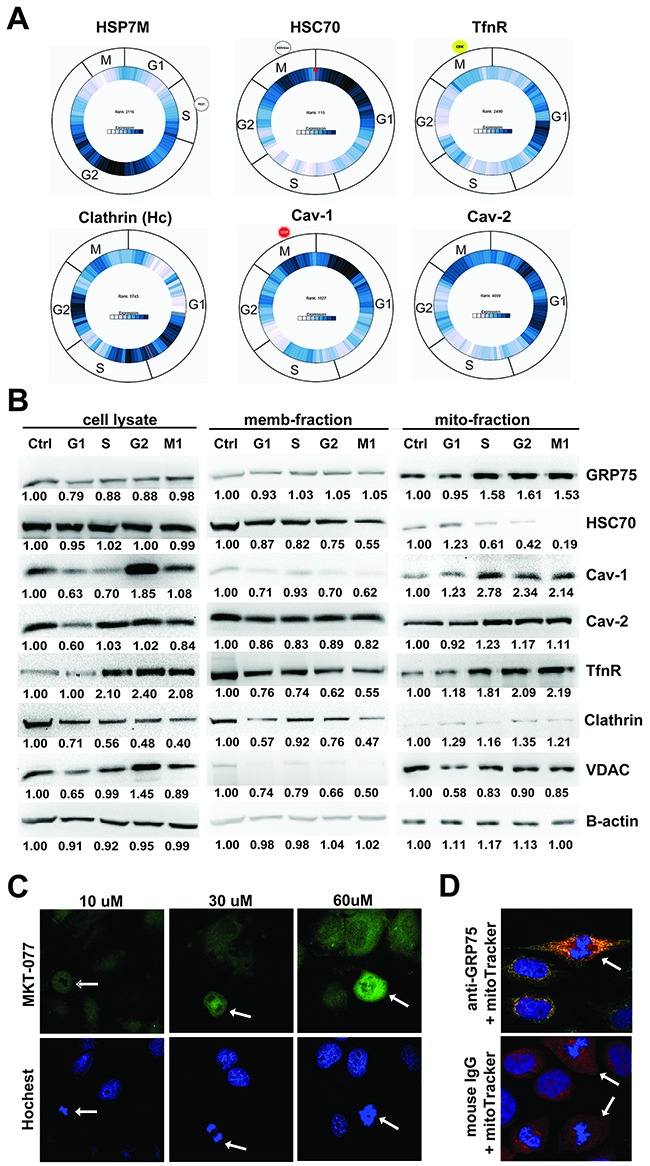
High expression of mitochondrial GRP75 in G2/M-phases **(A)** Predication of expression changes of HSP7M (GRP75 homologue), Hsc70, TfnR, clathrin, and caveolin-1/-2 in sub-phases of cell cycle usingCyclebase 3.0 analysis (dark blue content indexes the expression level of mRNA/protein during the cell cycle); **(B)** Western blot determined the expression level of those mentioned above in synchronized HeLa subcellular fractions. lysate-: whole cell lysate, memb-: membrane lysate, and mito-: mitochordrian lysate. Protein brands were quantified by Image J software and the expression ratio (compared to that of non-treated cells, Ctrl, set as 1) in the sub-phase is correspondingly marked below. Mitotic (arrow pointing) and interphase HeLa cells were stained by concentration-increased MTK-077 dye **(C)**, or co-stained by anti-GRP75 antibody (1:100) together with the mitochondrial marker mitoTracker®Red (300nM) **(D)**. Similar results were found in Cos-7 cells (Data not shown).

As membrane trafficking dynamically varies during cell division [33, 39] and our previous studies showed that GRP75 is essential to raft-associated endocytosis regulation [31, 32], we determined the expression change of GRP75 together with endocytic components in sub-phases of the cell cycle. As predicated by Cyclebase analysis, high-expression of the GRP75 homologue HSP7M frequently appeared in the G2-phase. However, high-expression of the CCV (clathrin-coated vesicle) dissociation factor HSC70 was mainly distributed in the G1-phase. High expression of clathrin was scarcely distributed in the M-phase, whereas highly-expressed TfnR appeared in the G1-phase. High expression of caveolin-1, the negative regulator of raft-dependent endocytosis, was frequently present in M- and G1-phases, which was in contrast to the high expression of raft endocytosis-dispensable caveolin-2 in the G1-phase (Figure 2A). Western blot analysis of cell fractions showed that the mitochondria-resident GRP75 was markedly higher expressed in S-, G2-, and M-phases than in the G1-phase. In contrast, the mitochondria- and membrane-associated HSC70 was sharply reduced in the M-phase. Although total cell lysate-derived and mitochondrial TfnR expression in S-, G2-, and M-phases doubled, membrane-resident TfnR expression was halved. The half reduction trend in these phases appeared similar to lysate-derived clathrin. Unexpectedly, expression of lysate-derived and mitochondrial caveolin-1 in G2- and M-phases doubled. However expression of membrane-resident caveolin-1 in the corresponding phases significantly decreased (Figure 2B). Furthermore, staining of cells by its rhodacyanine dye inhibitor MKT-077 at high concentrations or by its specific Ab, confirmed that mitochondria resident GRP75 was highly expressed in mitotic cells (Figures 2C, 2D). All these results indicate that GRP75 is highly expressed in the mitochondrial fraction in G2- and M-phases.

### Mito-trafficked GRP75 promotes cell cycle M-phase accumulation

**Figure 3:**
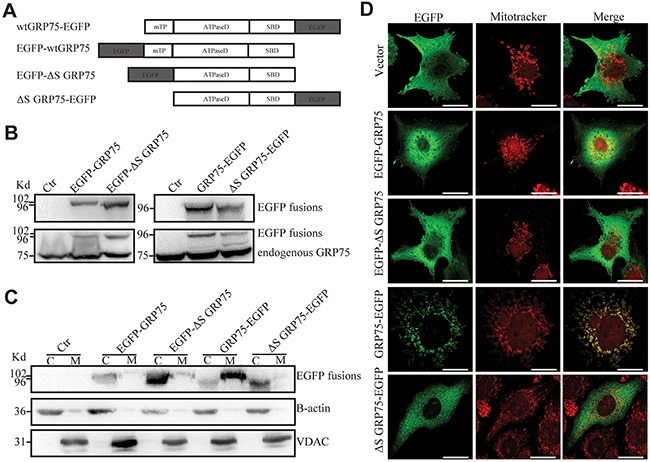
Signal peptide directed GRP75 expression-trafficked into mitochondria **(A)** Schematic representation of C-/N-terminal EGFP-fused GRP75 constructs with/without mitochondrial-targeting signal peptides. wt: wild type. mTP: mitochondrial-targeting signal peptide. ΔS: without signal peptide. ATPaseD: ATPase domain. SBD: substrate-binding domain. **(B)** HeLa cells were transfected with GRP75 constructs as listed above. Forty-eight hours post-transfection, the cell lysates were analyzed through SDS-PAGE followed by Western blotting with rabbit anti-GFP Ab and mouse anti-GRP75 Ab as described in Materials and Methods. **(C)** The cytoplasm (C) and mitochondria (M) fractions of transfected HeLa cells were analyzed by Western blotting with rabbit anti-GFP Ab (up row), anti-B-actin Ab (middle row), and anti-VDAC Ab (down row), respectively. **(D)** Transfected HeLa cells were first stained with mitoTrcker (300nM) for 10 min at 37°C for mitochondria labeling (red color). After fixation and permeabilization, cells were sequentially stained with rabbit anti-GFP Ab (1:500) and then with anti-rabbit AF488 (1:1000). The co-stained cells were viewed under a confocal microscope. Representative cell images show the contrast distribution of exogenous EGFP-fused proteins with mitochondria. Scale bar, 20 um.

**Figure 4:**
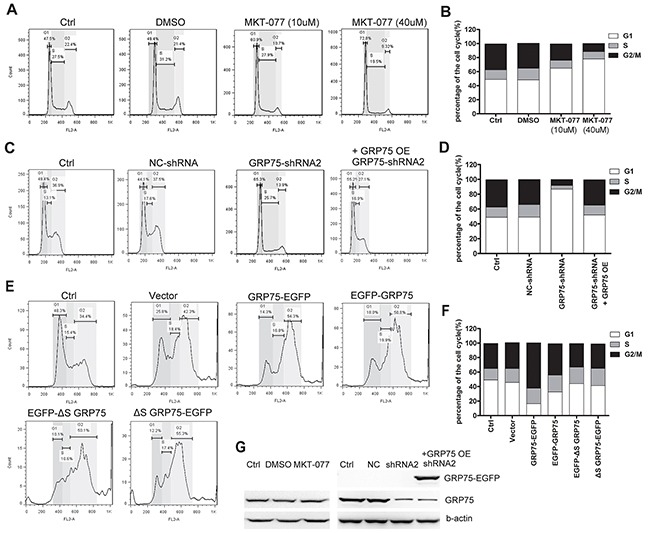
GRP75 expression promotes cell cycle enrichment in the M-phase **(A)** HeLa cells were treated with serially increasing doses of MKT-077 (5, 10, 20, and 40uM) for 24 hours prior to cell cycle analysis by propidium iodide staining and flow cytometry. **(C)** Cell cycle distribution was determined in HeLa cells stably transduced with lentivirus pLV-GFP-shGRP75RNAi (GRP75-shRNA), pLV-GFP-RNAi with negative control shRNA insertion (NC-shRNA), or further rescued by GRP75-EGFP plasmid overexpression. **(E)** HeLa cells were transiently transfected with GRP75 constructs as listed in Figure 3A. The cell cycle distribution was determined at 48 hours post-transfection. Untransfected (Ctrl) or pEGFP plasmid (vector) transfected cells were used for comparison. Representative FACS results are shown. **(B, D, F)** The graphs show the percentage of living cells in the various phases of the cell cycle after corresponding treatments **(A)**, or transfections **(C, E)**. Data are the average results from three independent experiments. **(G)** Western blot analysis of endogenous GRP75 level after MKT-077 blockage or shRNA knock-down in HeLa cells. Similar results were found in Cos-7 cells (Data not shown).

To determine the role of mitochondria resident GRP75 on the cell cycle distribution, we first constructed N-/C-terminal EGFP-fused GRP75 plasmids with/without a signal peptide for directed overexpression (Figure 3A). Bioinformatics prediction showed that only the GRP75-EGFP construct could be possible to target-express in mitochondria (Supplementary Table 1). Western blot results showed that the GRP75-EGFP construct was dominantly expressed in the mitochondria fraction with the predicted molecular weight (Figures 3B, 3C). This exclusive mitochondria-trafficking expression was further validated by confocal co-localization analysis based on mitoTracker Red labeling and anti-EGFP Ab staining (Figure 3D). After adjusting the transfected plasmids concentration to maintain an even expression of fusion proteins, flow cytometry analysis of the EGFP-positive cells showed that only GRP75-EGFP overexpression most significantly induced cell cycle accumulation in the M-phase (Figures 4E, 4F). Using the EGFP-labeled lentiviral shRNA expression system (shGRP75) that targeted different mRNA regions, GRP75 was substantially depleted (Supplementary Figure 2A), and cell cycle analysis showed that GRP75 depletion markedly arrested the cell cycle in the G1-phase (Figures 4C, 4D). We also treated both cells with the GRP75 inhibitor MKT-077, and found that MKT-077 treatment only significantly increased the HeLa cell population in the G1 phase in a dose-dependent manner (Figures 4A, 4B; Supplementary Table 2). These results suggest that GRP75 trafficking-expression in mitochondria promotes the accumulation of cancer cells in the M-phase.

### Mito-trafficked GRP75 inhibits CME but promotes CIE in the M-phase

**Figure 5:**
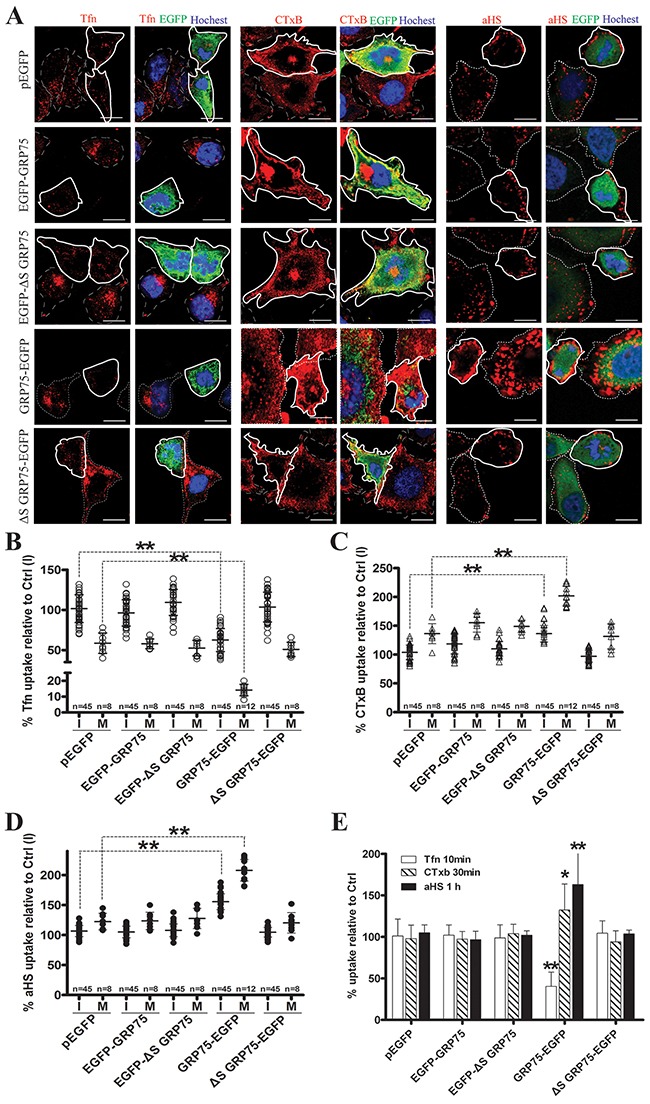
Mito-trafficked expression of GRP75 inhibits CME but promotes CIE HeLa cells were transfected with GRP75 constructs as listed in Figure 3A. Cells were quickly rinsed with PBS/0.5M NaCl, growth continued for 48h, then Tfn-AF647 (25μg/ml, 15min), CTxB-AF647 (10μg/ml, 30min), and αHS-AF647 complex (1:20, 1h) were respectively added at 37 °C. Cell uptake was determined by confocal imaging analysis, and representative images are shown in **(A)**. Scale bar: 20 um. **(B, C, D)** Scatterplots depict the uptake variability of indicated transfections in single cell populations. Uptake levels in the M (polygons with bold solid line) or I phase (polygons with thin dotted line) cells are quantified as described in ‘Materials and methods’, and the uptake of untransfected cells in the I phase, Ctrl (I), was set as 100% for comparison. At least 45 cells were counted for the I phase and ≥8 cells were counted for the M phase for each transfection. **(E)** The uptake levels of EGFP-positive cells collected from transfected populations were analyzed and quantified by FACS. 10,000 cells were counted per transfection in each experiment. Statistically significant differences compared with untransfected cells are shown: ** *P* <0.01, * *P* <0.05.

**Figure 6:**
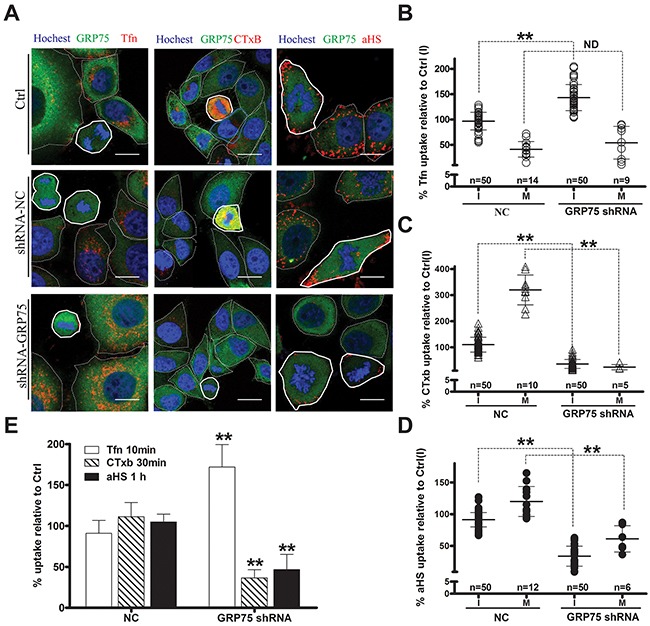
GRP75 knock-down promotes CME but inhibits CIE The uptake levels of Tfn-AF647, CTxB-AF647, and andαHS-AF647 complexes in GRP75-shRNA stably transduced HeLa cells were determined by confocal imaging analysis, and representative images are shown in **(A)**. Scale bar: 20 um. Scatterplots depict the uptake variability of indicated ligands, and the uptake levels in the M (polygons with bold solid line) or I phase (polygons with thin dotted line) cells are respectively quantified in **(B)**, **(C)** and **(D)** with comparison to that of untransfected cells in the I phase as Ctrl (I). At least 50 cells were counted for the I phase and ≥5 cells were counted for the M phase for each transfection. **(E)** The uptake levels of EGFP-positive shRNA cell populations were analyzed and quantified by FACS. 10,000 cells were counted per sample in each experiment. Statistically significant differences compared with negative control cells (NC-shRNA) are shown: ** *P* <0.01, ND: no difference.

**Figure 7:**
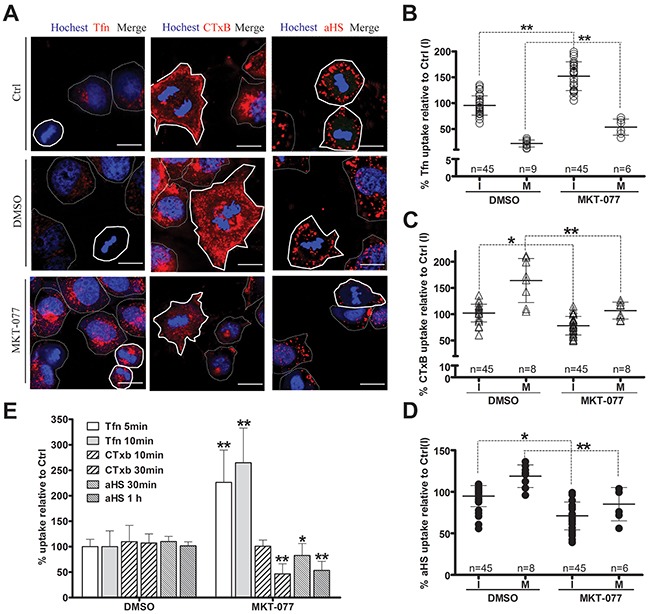
GRP75 targeting by MKT-077 promotes CME but inhibits CIE HeLa cells were treated with concentration-increased MKT-077 (5, 10, 20, and 40uM) for 24 hours, and then incubated respectively with Tfn-AF647, CTxB-AF647, and αHS-AF647 complexes for uptake measurement as described above. Representative confocal images are shown in **(A)**. Scale bar: 20 um. Scatterplots depict the uptake variability in M (polygons with bold solid line) or I phase (polygons with thin dotted line) cells, and the uptake levels compared to that of untreated cells in the I phase (Ctrl(I)) are summarized in **(B)**, **(C)** and **(D)**. At least 45 cells were counted for the I phase and ≥6 cells were counted for the M phase for each treatment. **(E)** Ligands uptake in MKT-077 treated cells were determined by flow cytometry analysis, and the uptake levels are quantified in **(E)**. 10,000 cells were counted per sample in each experiment. Statistically significant differences compared to untreated cells (Ctrl) are shown: **P <0.01, *P <0.05.

Since endocytosis variation is pronounced during cell division, the impact of the cell cycle-dependent expression of GRP75 on the endocytosis pathway was then examined. Confocal imaging analysis of EGFP-positive cells showed that GRP75-EGFP overexpression markedly increased the uptake of CTxB (average increases of 22% in the I-phase and of 59% in the M-phase), increased the uptake of the αHS complex (average increases of 49% in the I-phase and of 100% in the M-phase), but significantly decreased the uptake of Tfn (average decreases of 30% in the I-phase and 41% in the M-phase), and all induced more uptake changes in the mitotic phase than that in the interphase (Figures 5A-5D). In contrast, overexpression of other GRP75-fused constructs resulted in little changes to the uptakes. These GRP75 mitochondria-localization-induced effects on cell cycle dependent endocytosis were similarly observed in flow cytometry quantification results (Figure 5E). To further confirm this GRP75-dominated effect, EGFP-labeled shGRP75 stable cell lines were used. Confocal and flow cytometry quantification analyses of these cells showed that GRP75 depletion significantly increased the uptake of Tfn in the interphase (average increments 45% and 78%, respectively), more markedly decreased the uptake of CTxB in the mitotic phase (average decrements 275% and 78%, respectively) than that in the interphase (average decrements 65% and 42%, respectively), and limited the uptakes of the αHS complex more in the mitotic phase (average decrements of 61% and 54%, respectively) (Figure 6, Supplementary Figure 6). Further treatment of cells with the GRP75 inhibitor MKT-077 obtained similar results (Figure 7). These results together with the aforementioned data suggest that mitochondria-trafficked GRP75 significantly promotes CIE but inhibits CME primarily in the M-phase.

### GRP75 targeting enhances G1 phase-privileged nanomicrosphere uptake

**Figure 8:**
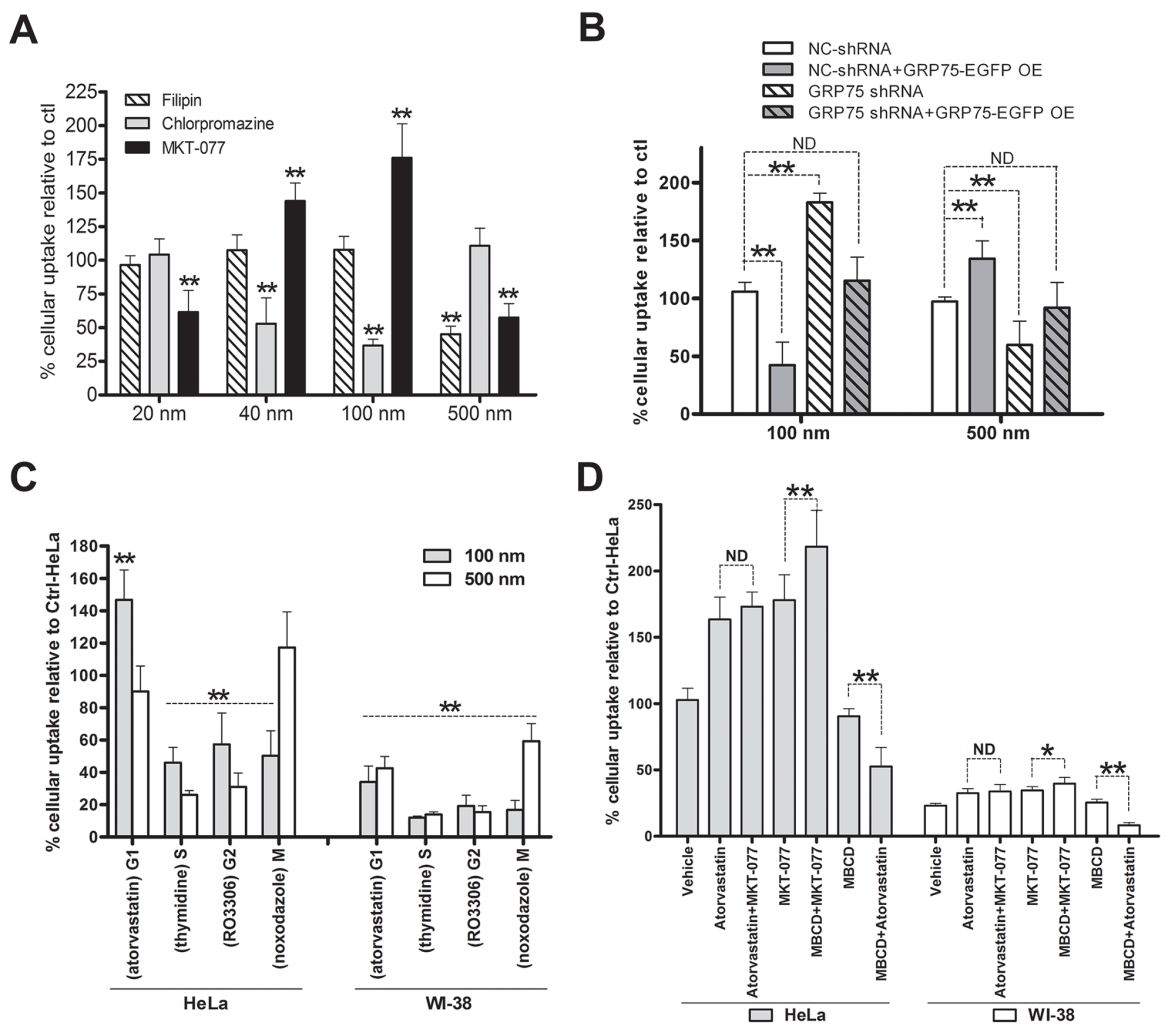
GRP75 targeting by MKT-077 enhances G1-phase-privileged uptake of 100 nm nanomicrospheres **(A)** Effect of CME, CvME, and GRP75 inhibitions on the uptake of size-defined nanomicrospheres. HeLa cells were incubated respectively with filipin (5μg/ml), chlorpromazine (10μg/ml), or with MKT-077 (40uM) in serum-free medium for 24h. Subsequently, different sizes of fluorescent microspheres (1:300, v/v) were added and incubated for 12h in the presence of inhibitors. The uptakes of microspheres in cells were determined by flow cytometry analysis. The fluorescent intensity of nanoparticles in DMSO treated cells (Ctrl) was set as 100%. **(B)** Effect of GRP75 knock-down and overexpression on the uptake of nanomicrospheres. GRP75-shRNA stably transduced HeLa cells (GRP75-shRNA) were transfected with the wtGRP75 plasmid for overexpression (GRP75-OE) for 12h. After extensive washing and continued growth for 18h, 100nm or 500m fluorescent microspheres (1:300) were added to the cells for 12h incubation and processed for flow cytometry analysis. The fluorescent intensity in cells was compared to that of the negative control (NC-shRNA). **(C)** Effect of arrested cell cycle phases on the uptake of nanomicrospheres. HeLa and WI-38 cells were synchronized at different cell cycle phases as described above. Fluorescent microspheres of 100 and 500nm sizes were respectively added (1:300) and incubated for 12 h in the presence of chemical inducers. The uptakes of microspheres were determined by flow cytometry and intracellular the fluorescent intensity of untreated HeLa cells was set as 100 % (Ctrl-HeLa). **(D)** Effect of MKT-077 combined with atorvastatin or MBCD on the uptake of 100 nm fluorescent microspheres. HeLa and WI-38 cells were respectively incubated with the mixtures of MKT-077 and atorvastatin, or MKT-077 and MBCD, or MBCD and atorvastatin. The uptakes of nanomicrosphere (1:300) in single or combined treated cells were determined by flow cytometry and compared to that of untreated HeLa cells (Ctrl-HeLa). *0.01 < *P* < 0.05, **P < 0.01, ND: no difference.

To examine whether GRP75-induced cell cycle-privileged endocytosis can be capitalized, we tested its inhibitor to improve the delivery efficiency of nanomicrospheres sized from 20 nm to 2um in diameter. Cytometry quantification results showed that HeLa cells took up fluorescent microspheres in both a time- and size-dependent manner (Supplementary Figure 3). Little uptake was seen for particles sized 2 μm. Pretreating the cells with the CME inhibitor chlorpromazine significantly decreased the uptake of microspheres of size 40 nm and 100 nm to 52±9% and 38±4%, respectively, whereas the uptake of those sized 20 nm and 500 nm was not significantly altered. Pretreating the cells with the caveolae-disrupting agent filipin only reduced the uptake of 500 nm microspheres to 45±6%, but brought no uptake changes to other sizes of microspheres. In contrast, MKT-077 treatment markedly enhanced the uptakes of 40 nm and 100 nm microspheres to 144±11% and 178±21%, respectively, but significantly impaired the uptakes of 20 nm and 500 nm microspheres to 64±12% and 55±10%, respectively (Figure 8A). Further checking the uptakes in shGRP75 stable cell lines showed that internalized microspheres of 100 nm were markedly increased, which was in contrast with the reduced uptake of 500 nm microspheres. The distinct uptake changes of microspheres induced by GRP75 knock-down were efficiently rescued by exogenous GRP75 overexpression (Figure 8B). These results indicate that cells internalized 40 nm to 100 nm microspheres via the CME pathway, internalized 500 nm microspheres mainly via the CvME pathway, and that targeting GRP75 enhanced CME of nanomicrospheres sized from 40 nm to 100 nm.

Since nanoparticle uptake is influenced by the cell cycle phase [40, 41], the endocytosis pathway variation in cell cycle stages may lead to the differential uptake of certain particles. Comparing the uptakes in synchronized HeLa cells, it was found that 100 nm microspheres were most dominantly concentrated in the cell cycle G1-phase, whereas 500 nm microspheres were mainly accumulated in G1- and M-phases (Figure 8C). These well agree with the afore-obtained results, suggesting that enhanced uptake of 100 nm microspheres may be largely due to the G1-phase retention effect. To further examine whether GRP75 targeting can potentiate this effect, we treated the cells with different drugs combinations. Unexpectedly, MKT-077 treatment did not augment atorvastatin-induced G1-phase uptake-enhancement of 100 nm microspheres. MBCD was frequently shown to deplete cholesterol from cell membranes thereby altering the transport of many drug molecules through diffusion or receptor meditated uptake [42]. When cells were treated with MKT-077 combined with MBCD, the uptake of 100 nm microspheres increased dramatically to 220±21% as compared to either agent alone (Figure 8D). On the contrary, no synergistic effect but counteraction phenomena was observed in MBCD combined with atorvastatin treatment. Interestingly, these contrast effects induced by either single drug pretreatment or by MKT-077 combined with MBCD/atorvastatin pretreatment were similarly observed in mito-GRP75 low-expressed WI-38 cells (Figures 8D, Supplementary Figure 4), where the whole uptake of 100 nm microspheres was fairly low (Figure 8D).

## DISCUSSION

The conventional concept of glucose-regulated proteins (GRPs) as protein folding chaperones was updated by discoveries that GRPs promote tumor proliferation, metastasis, drug resistance, and immunotherapy. As a mitochondria matrix dominant chaperone highly expressed in cancer cells and contributing to carcinogenesis, GRP75 has been identified as an important oncotarget, and its inhibition could dramatically improve cancer patients’ responses to chemotherapies [43, 44]. Recent advances showed that GRP75 has functions that are distinct from those of the related heat shock proteins. It can be actively translocated to other cellular locations and therefore assume novel functions that control intracellular trafficking, modify the immune response, and regulate viral release [31, 32, 45, 46]. We previously demonstrated the presence of GRP75 at the HeLa cell surface as well as its enrichment in endocytic vesicles. Importantly, we provided evidence for a functional role of GRP75 in HSPG ligand-induced vesicular transport of nanoparticle payloads (scFv-αHSM, scFv-αHSF, and Tat-DNA complexes) through Cdc42-dependent, membrane raft endocytosis [31]. More recently, we demonstrated that the uptake of the HSPG-dependent macromolecular complex occurs through the CIE pathway, which is regulated by upstream GRP75 in HeLa cells. This endocytosis regulation is largely mediated by GRP75 in the mitochondria and is essentially determined by its ATPase domain. Overexpression of GRP75 or its ATPaseD in mitochondria stimulates RhoA and Cdc42 concurrent activation and enhances the formation of stress fibers and filopodia, collectively resulting in the promotion of CIE but the inhibition of CME [32]. In the present study, we further demonstrated that GRP75 is highly expressed in the mitochondrial fraction of mitotic HeLa and Cos-7 cells. This mitochondria-trafficked GRP75 expression, not only significantly arrests cancer cells in the cell cycle M-phase, but also promotes CIE and simultaneously inhibits CME primarily in this arrested M-phase. Therefore, we claim that the GRP75 moonlighting function is a cell cycle controller and endocytosis regulator in cancer cells. More importantly, this moonlighting function of GRP75 can be utilized to enhance the CME of nanomicrospheres sized from 40 nm to 100 nm through the GRP75 targeting/inhibition-induced G1-phase retention effect.

Different from the prevailing dogma that endocytosis is shut down during cell division, our present data showed that CME was markedly reduced but not completely halted in the cell cycle M-phase (Figures 1A, 1B, 1E). In mitotic cells, we detected decreased expression of clathrin in the total lysate and reduced CCV dissociation factor HSC70 in the membrane fractions, which sharply contrasts with the fold-increased total TfnR and decreased membrane-resident TfnR (Figure 2B). The distinct distribution of these endocytosis components during the cell cycle suggests that strong reduction of the Tfn uptake observed in the M-phase is not only due to the decreased CME but also the attenuated TfnR recycled from the cell surface, which supports the recent consensus that CME is inhibited during mitosis [33, 34, 36]. Although it is hard to explain why caveolin-1 expression between membrane-fractions and the total lysate presents a contrary distribution in the M-phase (Figure 2B), the continous even up-regulated uptake of the CvME marker CTxb in mitotic cells (Figures 1A, 1C, 1F) indicates that CIE activity may be required for plasma membrane remodeling when cells undergo division. This is somehow similar to the previous report that EGF internalization in mitotic cells occurs much slower at the early stage of mitosis but the total amount of internalized EGF approaches that seen in interphase cells [47]. However, we observed that CME dominated in the I-phase versus up-regulated CIE in the M-phase which reveals that there is an endocytosis balance or shift during the cell cycle.

Although membrane-associated GRP75 is dispersed during the cell cycle, mitochondrion-resident GRP75 is highly enriched during G2/M-phases (Figure 2B). The cell cycle specific expression and differential mitochondria localization together with its highly expression-induced mitotic phase arrest (Figure 4), collectively indicate the role of GRP75 in cell cycle control. However, the underlying mechanism whereby GRP75 controls cell cycle distribution was not the focus of our present study. Work from other labs contributes a lot on this aspect. In MEK/ERK-activated cancer cells and hematopoietic stem cells, GRP75 depletion/targeting-induced cell cycle arrest was accompanied by increased expression of cyclin-dependent kinase inhibitor p21^CIP1^, tumor suppressor p53, and decreased expression of cyclin-dependent kinase inhibitor p27^KIP1^, S-phase transcription factor E2F-1, and Rb phosphorylation, indicating that GRP75 may regulate the cell cycle via cyclin-dependent kinas/TP53/Rb signaling [23, 26, 28, 30, 48]. Notably, another study on cell cycle arrest experiments found that HSP70 specific inhibitor PES-Cl treatment could effectively inhibit cyclin B degradation and APC/C (anaphase promoting complex/cyclosome) activity, which always resulted in G2/M arrest in cancer cells [29]. As several HSP70 family members are required for CDK1/cyclin B complex assembly, whether GRP75 is also involved in this complex activity is worthy of future research.

Mitochondria are highly dynamic organelles which always transport to special cellular regions to meet energy needs. Because GRP75 is indispensable to mitochondrial morphology and bioenergetic maintenance, from the perspective that endocytosis and cell division are energy-dependent cellular behaviors, GRP75 localized in mitochondria should be essential to both processes. We previously found that vesicle-enriched endogenous GRP75 also appears on the cell surface and exogenous GRP75 trafficking-expressed in mitochondria could up-regulate CIE [31, 32]. In this study, we further strengthened this aspect by showing the contributions of mitochondria-trafficked GRP75 on cell cycle M-phase accumulation and endocytosis shift (Figures 4, 5). Such subcellular localization-determined moonlighting function was also reported on other endocytosis regulators and chaperone proteins: (1) Cdc42 GTPase is the master regulator of clathrin- and dynamin-independent endocytosis. Silencing of its specific GEF Intersectin 2 can attenuate Cdc42 activation but also disrupt the correct orientation of the mitotic spindle during epithelial morphogenesis [49]. (2) A sub-fraction of cytoplasmic chaperone HSP70 was shown to cluster in lipid raft areas, which enhances CIE through oligomerisation and clustering on the melanoma cell surface [50]. (3) Significant amounts of endoplasmic reticulum resident chaperone calreticulin were found in physical association with MHC I molecules on the activated T cell surface [51]. (4) Another endoplasmic reticulum chaperone GRP78 was found to act as a binding receptor for caveolae-dependent endocytosis of dentin matrix proteins [52]. In light of the dynamic transport of mitochondria to the cell membrane, these observations together with our present findings suggest that M-phase-enriched mito-GRP75 may maintain the stability of lipid raft-associated membrane flow structures, which promotes CIE but inhibits CME in mitotic cancer cells.

Nanoparticles are considered a primary vehicle for targeted therapies because they can pass biological barriers and enter and distribute within tissue cells by energy-dependent pathways. The big problem is that the uptake of nanoparticles by cells is not only affected by their properties, such as size and surface charge, but also influenced by the cell cycle phase. Some studies have shown that the cellular uptake efficiency of nanoparticles during the cell cycle is ranked as follows: G2/M > S > G0/G1 [40, 41, 53]. Utilizing this G2/M phase retention effect, researchers elevated the uptake of nanoparticles in tumors by different chemotherapeutic pretreatments [54, 55]. Despite gaining intracellular delivery of nanoparticles, the final effects of tumor suppression *in vivo* are ambiguous. One reason for this is that at different stages of the cell cycle, the dominant endocytosis pathway can vary leading to the differential uptake of certain particles. Another reason is that tumor growth is characterized by an exponential proliferation phase and a second non-proliferating phase, in which cancer cells are always dormant in the cell cycle G0/G1 phase due to nutrient deprivation or hypoxia. Since CME is suppressed during mitosis but dominant at the interphase of cell cycle (Figure 1), and cancer cell internalize 40 nm to 100 nm nanomicrospheres via the CME pathway (Figure 8A), one solution is the use of combined therapies, in which one component accumulates the cell cycle at the G1 phase, and another delivers the therapeutic drugs through CME. As MKT-077 is a cancer cell-selective small molecule inhibitor, our present initiative based on the utilization of the GRP75 targeting-induced G1-phase-privileged CME route is a simple approach to enhance the delivery efficiency of such kind of nanoparticles. Although we only have promising data on improving certain sizes of nanomicrospheres delivery (Figure 8D), more work is needed to consummate this moonlight function targeting approach. This should at least include checking the delivery efficiency of ligands (e.g. αHS Ao4B08, Tfn, TAT)-modified active targeting nanomicrospheres *in vitro* and *in vivo*. Such experimental work has begun in our lab.

## MATERIALS AND METHODS

### Cell culture, nanoparticles, antibodies, and reagents

HeLa, Cos-7, WI-38, and 293T cell lines obtained from the Type Culture Collection of Chinese Academy of Sciences (P.R. China), were maintained in Dulbecco's modified eagle's medium (DMEM) containing 10% (v/v) fetal bovine serum. Red fluorescent (580/605) labeled, carboxylate-modified microspheres were obtained from Thermo Fisher without further modification. The sizes of fluorescent nanoparticles were 20, 40, 100, 500, and 2000 nm in diameter (F8786, F8793, F8801, F8812, and F8826, respectively). The mouse anti-GRP75 antibody (Ab) (SC-133137), anti-Hsc70 Ab (sc-7298), anti-TfnR Ab (sc-32272), anti-clathrin Ab (sc-12734), anti-caveolin-1 Ab (sc-53564), and anti-caveolin-2 Ab (sc-7942) were obtained from Santa Cruz. The rabbit anti-GFP Ab (D110008), anti-B-actin Ab (sc-7210), anti- voltage-dependent anion channel 1(VDAC) Ab (D151112), thymidine (A500943), nocodazole (A606391), colcomid (A600322), and mitochondria/cytoplasm isolation kits (C500049, C500051) were obtained from Sangon Biotech. The mitochondria-selective probe mitoTracker®Red (M7512), Tfn-alexa fluor (AF) 647 (T-23366), CTxB-AF647 (C-34778), goat anti-mouse Ab-AF488 (A-11001), anti-mouse IgG-AF647 (A32728), anti-rabbit Ab-AF488 (A-11070), and Lipofectamine 3000 were obtained from Life technologies. The mouse anti-VSV Ab P5D4 (V5507), MKT-077 (M5449), atorvastatin (PZ0001), RO3306 (SML0569), Hochest33342, propidium Iodide (P4170), RNAseA (R6513), polybrene (H9268), and puromycin (P7255) were obtained from Sigma.

### Plasmid constructs for knock-down and overexpression

EGFP N-/C-terminal fused GRP75 plasmids with/without mitochondrial targeting signal peptide (mTP) (wtGRP75-EGFP, ΔSGRP75-EGFP, EGFP-wtGRP75, and EGFP-ΔSGRP75) were as previously constructed [32, 56]. For gene overexpression, recombinant GRP75 plasmids were transient transfected into 60-70% confluent cells by Lipofectamine®3000 according to the manufacturer's instructions. The GFP-tagged pLV-RNAi vector system was obtained from Biosettia for long-term gene silencing. Three independent GRP75-targeting shRNA constructs were generated by ligation of annealed oligonucleotides, which target different regions of human GRP75 mRNA (shRNA#1: GCAGTTGTTGGTATTGATT, shRNA#2: GCTCATGGGAAATTGTATT, and shRNA#3: GCCCTATCTTACAATGGAT), into the pLV-H1-EF1α-GFP-Puro plasmid (SORT-B29), respectively. For lentivirus production, 293T cells were co-transfected by recombinant pLV-RNAi plasmids together with Gag-Pol, Rev, and VSV-G packaging plasmids, as described in their manual. Viral supernatants were collected after 72 h and mixed with polybrene (4 ug/ml) before use. Viral titers were determined by scoring cells expressing green fluorescent protein (EGFP). The optimal puromycin concentration (3μg/ml) was used to select stably transduced cell lines. Specific overexpression and knockdown of the GRP75 protein were confirmed by Western blotting.

### Culture synchronization and cell cycle analysis

Synchronizing cells from different phases of the cell cycle was performed by the protocol previously described [57]. Briefly, cells were treated with serum-free/limited medium (G0/G1 phase), or treated with for HMG-CoA reductase inhibitor atorvastatin (G1 phase), or treated with double thymidine block procedure (S phase), or treated with CDK1 inhibitor RO3306 (G2 phase), or treated with nocodazole/colcomid (M phase) for indicated times as described in the legend of Supplementary Figure 1. To assess the cell cycle distribution, all the above cells were collected and fixed in 70% ethanol overnight. After removal of the ethanol, samples were washed three times with PBS, and then incubated with RNase A at 4°C for 30 min. Next, samples were stained with propidium iodide (50μg/ml) and evaluated by a flow cytometer. To assess the cell cycle distribution after GRP75 knock-down or overexpression, transfected cells were sorted by FACS Calibur (BD) with a gate that separated the EGFP-positive cells for cell cycle analysis as described above. The subsequent analysis was conducted by FlowJo software.

### Uptake measurement by flow cytometry analysis

To measure the uptakes, transfected or synchronized cells was first washed twice with serum-free medium to reduce paracrine effects. Only attached live cells were exposed to check the binding and uptake of Tfn-AF647 (25μg/ml), CTxB-AF647 (10μg/ml), αHS-AF647 complex (1:20), and fluorescent nanospheres (1:300) in incomplete medium as previously described [31, 32]. Binding incubation with these ligands was performed for 30 min on ice. Cells were then washed twice with PBS and immediately analyzed on the FACSCalibur instrument integrated with Cell-Quest software. For uptake measurement, cells were incubated with Tfn-AF647 (15min), CTxB-AF647 (30min), αHS-AF647 complex (1h), and fluorescent nanospheres for indicated times at 37 °C, and then 0.4% (w/v) Trypan Blue in PBS was added to quench cell surface-associated fluorescence. Samples were washed twice with PBS/0.5M NaCl, harvested with trypsin/EDTA, resuspended with PBS, and intracellular fluorescence was immediately detected using the FACSCaliber flow cytometer.

To compare the uptake of a synchronized culture to that of a non-synchronized one, care was taken to ensure that a similar number of cells were prepared for fluorescent ligands/nanoparticles uptake. In addition, prior to addition of fluorescent ligands/particles, various inhibiting conditions were achieved in cells via 24 h incubation with serum-free media containing chlorpromazine (10mg/mL), filipin (5mg/mL), atorvastatin (1mg/mL), methyl-B-cyclodextrin (10mM), or MKT-077 at a concentration as described in the text. Stock solutions of these drugs were made in DMSO, diluted in PBS, and the final concentration of DMSO was less than 0.4%.

### Uptake measurement by confocal imaging analysis

Cells in chamber slides were first washed twice with serum-free medium, and then incubated with fluorescent labeled ligands/nanoparticles as described above at 37 °C. After rinsing twice with PBS/0.5M NaCl to remove cell surface-associated ligands, cells were fixed in 3.7% (w/v) paraformaldehyde for 10 min. Honest33342 (1:1000) nuclear staining was performed after cell permeabilization with 0.1% Triton X-100. The cells were mounted, observed, and images were acquired using an Olympus FV 1000 laser scanning confocal microscope equipped with 63×/1.4 N.A. and 100×/1.44 N.A. oil immersion lens. Sixteen-bit z-series of confocal sections (step size = 0.42 μm for 63× objective and 0.29 μm for 100×objective) were acquired in the photon-counting mode, and these acquisition parameters were kept identical across all samples. For image presentation, eight-bit maximal projections of the z-series were created using ImageJ software, and the brightness was adjusted across the entire image using Volocity software. The quantification details of these fluorescent ligands/particles were previously described[32].

For labeling of mitochondria and immunostaining of EGFP-fused proteins, transfected cells were incubated with 300nM mitoTracker at 37°C for 40min, then washed twice with PBS/0.5M NaCl, fixed in 3.7% formaldehyde for 10min at room temperature, penetrated with 0.1% TritonX-100 for 5min at room temperature, sealed with 10% goat serum, and immunostained with anti-GFP antibody (1:1000), followed by staining with goat anti-rabbit Ab-AF488 (1:1000), and then cells were mounted for confocal imaging.

### Cell fractionation and western blotting

The cytosol, membrane, and mitochondrial fractions of synchronized cells were extracted by the differential centrifugation method as described in the kit description. The protein concentration of the samples in lysis buffer (50 mM Tris-HCl, pH 8, 150 mM NaCl, 1% NP-40, 0.5% sodium deoxycholate, and 0.1% SDS) was measured using the BCA assay, resolved by SDS-PAGE, blotted onto PVDF membranes, and then analyzed with different Abs diluted as follows: rabbit anti-GFP Ab (1:2000), anti-B-actin Ab (1:1000), anti-VDAC Ab (1:2000), mouse anti-GRP75 Ab (1:1000), anti-Hsc70 Ab (1:500), anti-caveolin-1 Ab (1:500), anti-caveolin-2 Ab (1:500), anti-TfnR Ab (1:500), and anti-clathrin Ab (1:500).

### Bioinformatics prediction

Online programs of WOLF PSORT (http://genscript/wolf-psort.html), TargetP 1.1 (http://cbs.dtu.dk/services/TargetP), and Mitoptot II (https://ihg.gsf.de/ihg/mitoprot.html), all three based on predicting N-terminal mTP and calculating the probability of proteins imported into the mitochondria, were used to predict the subcellular localization of EGFP-fused GRP75 proteins. Online database Cyclebase 3.0 [58], a multi-organism database on cell-cycle regulation and phenotypes, inside which mRNA and protein expression information based on high-content screens and model organism data integrated from genome-wide, cell-cycle-related experiments, was used to predict the expression level of GRP75 in sub-phases of the cell cycle.

### Statistical analyses

Confocal microscopy, flow cytometry, and Western blotting data were derived from at least three independent experiments. All of the data obtained from the experiments were analyzed and are presented as the mean ± SD. For two-sample comparisons against the controls, unpaired Student's t-tests were used unless otherwise noted. One-way analysis of variance with Dunnett's multiple comparison was used to evaluate the statistical significance of at least three groups of samples. Graphs were created using GraphPad Prism 5 software.

## SUPPLEMENTARY MATERIALS FIGURES AND TABLES



## Abbreviations

Ab: antibody
AF647: alexa fluor dye 647
αHS: anti-heparin sulfate single chain antibody
CME: clathrin-mediated endocytosis
CIE: clathrin-independent endocytosis
CvME: caveolae-mediated endocytosis
CTxB: cholera toxin B subunit
EGFP: enhanced green fluorescent protein
GRP: glucose-regulated protein
HSPG: heparan sulfate proteoglycan
mTP: mitochondrial-targeting signal peptide
ΔS: without mitochondrial-targeting signal peptides
Tfn: transferrin
TfnR: transferrin receptor
