# Supplementary material for: Mitochondria chaperone GRP75 moonlighting as a cell cycle controller to derail endocytosis provides an opportunity for nanomicrosphere intracellular delivery
Source: Oncotarget. 2017 Apr 19;8(35):58536–52. doi: 10.18632/oncotarget.17234 (PMC5601673; doi:10.18632/oncotarget.17234)
Supplement: Supplementary file 1 [file oncotarget-08-58536-s001.pdf]

Mitochondria chaperone GRP75 moonlighting as a cell cycle controller to derail endocytosis provides an opportunity for nanomicrosphere intracellular delivery

SUPPLEMENTARY MATERIALS

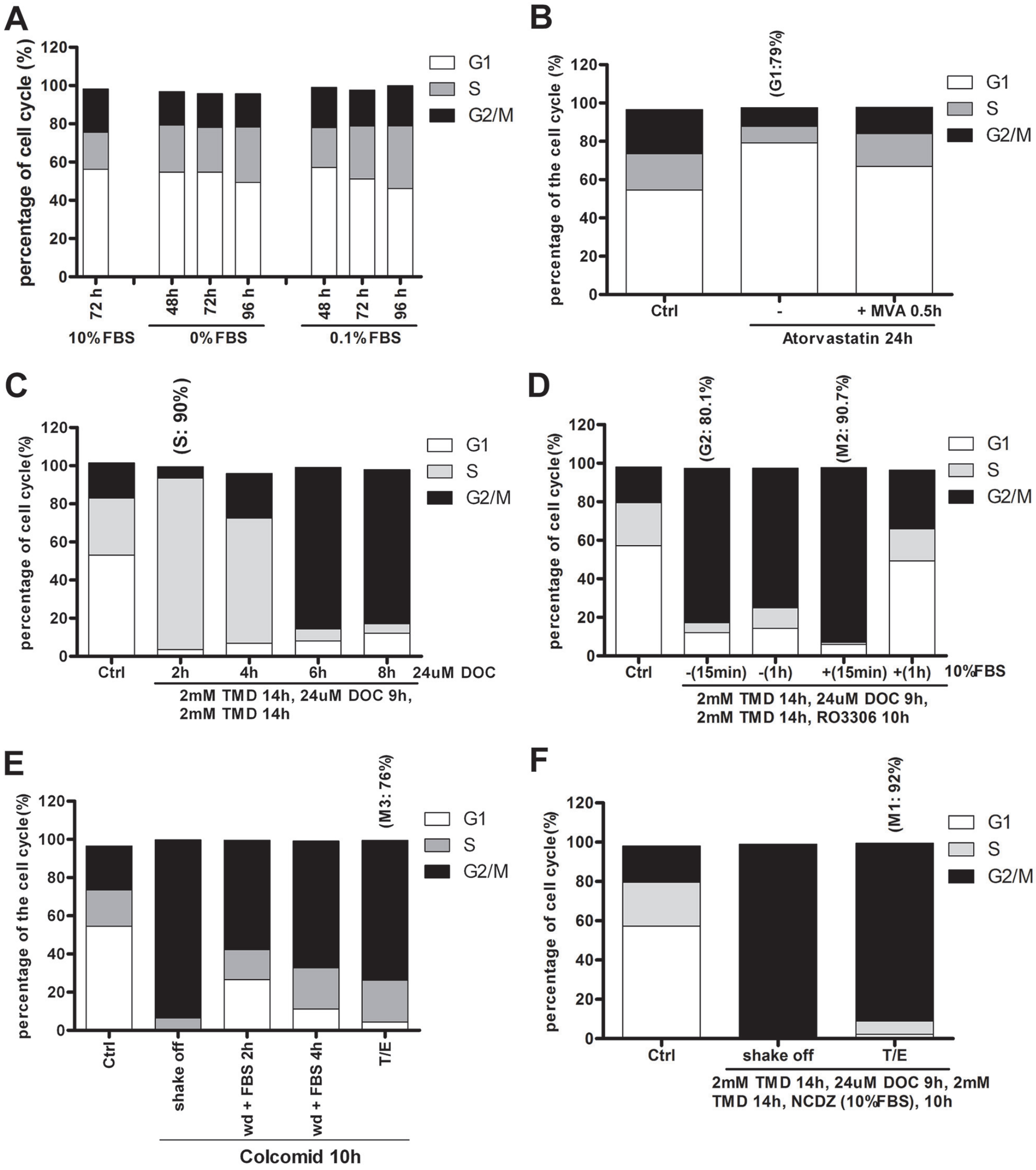

Supplementary Figure 1: Cell cycle arrest induced by drugs treatment. Serum-starved or drugs-treated HeLa cells were collected by T/E digestion and transferred for cell cycle determination by flow cytometry analysis. **(A)** No G1-phase arrest was reached after 0% or 0.1% serum starvation; **(B)** G1-phase arrest was maximally reached after atorvastatin (20uM) incubation without mevalonic acid (60uM) further incubation; **(C)** S-phase arrest was maximally reached after two rounds of thymidine (2mM) incubation followed by deoxycytidine (24uM) incubation for 2 hours; **(D)** Maximal G2-phase arrest was reached after two rounds of hymidine incubation followed by RO3306 (10uM) incubation, then slippage to the M-phase (M2) was reached after 15 min 10% FBS stimulation; **(E)** M-phase arrest (M3) was largely reached after colcomid (0.1uM) incubation followed by shaking to get rid of suspended cells; **(F)** M-phase arrest (M1) was maximally reached after sequentially added thymidin and deoxycytidine followed by noxodazole (0.1ug/mL) incubation for two rounds. Similar results were found in Cos-7 cells (data not shown).

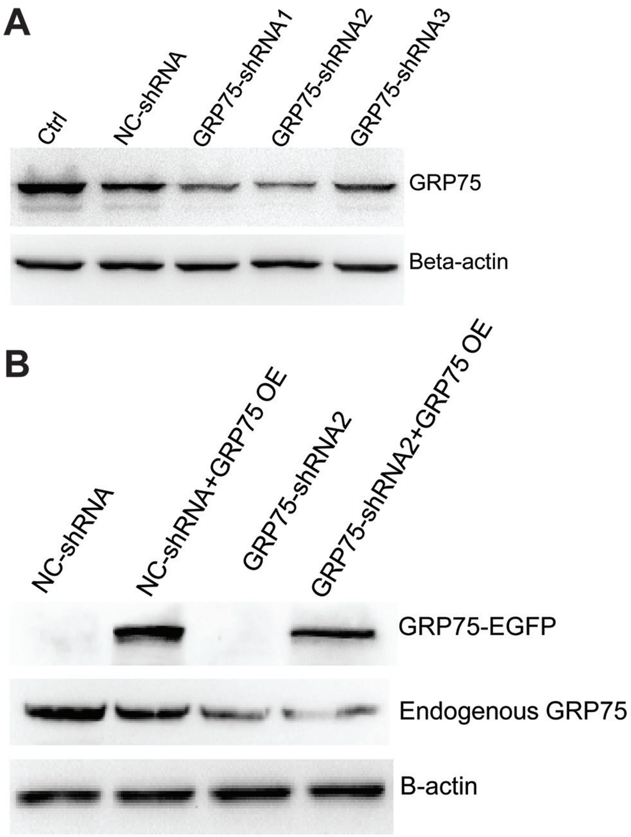

Supplementary Figure 2: Checking GRP75 interference level after knockdown and/or overexpression. (A) Equal amount (10ug) of cell lysate from GRP75-shRNA stable cell lines were separated on SDS-PAGE and subjected to Western blot analyses. **(B)** GRP75-shRNA2 stable cells were transiently transfected with GRP75 constructs for overexpression, and equal amount of cell lysates were blotted with mouse anti-GRP75 Ab (middle row), rabbit anti-GFP Ab (upper row) and anti-B-actin (lower row), respectively.

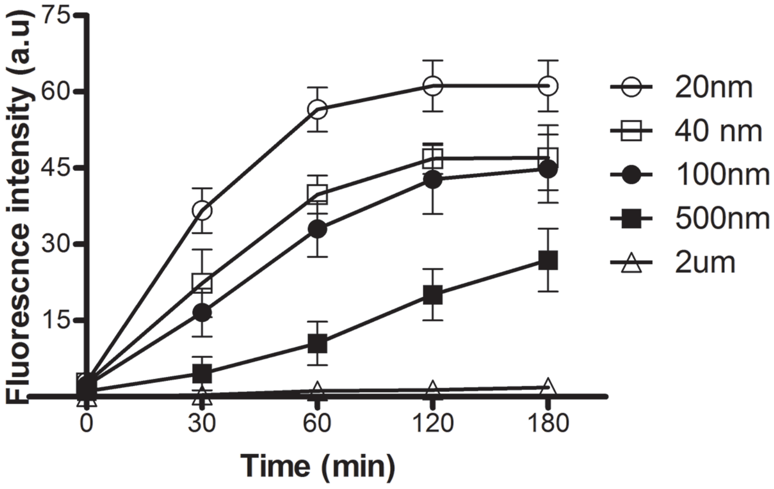

Supplementary Figure 3: Cellular evaluation of the dynamic uptake of nanomicrospheres. HeLa cells were incubated with size-differed fluorescent microspheres (1:300) for indicated time periods. Subsequently, cells were trypsinized, collected, unbound microspheres were washed off, and cells were processed for flow cytometry analysis. Cells incubated with serum-free media were used to calculate time zero. Results show that small sized microspheres had a quicker and higher uptake in intracellular fluorescent intensity compared to large sized microspheres. Uptake of fluorescent microspheres smaller than 500nm reached a plateau after 2–3 hours, indicating saturated binding on HeLa cells.

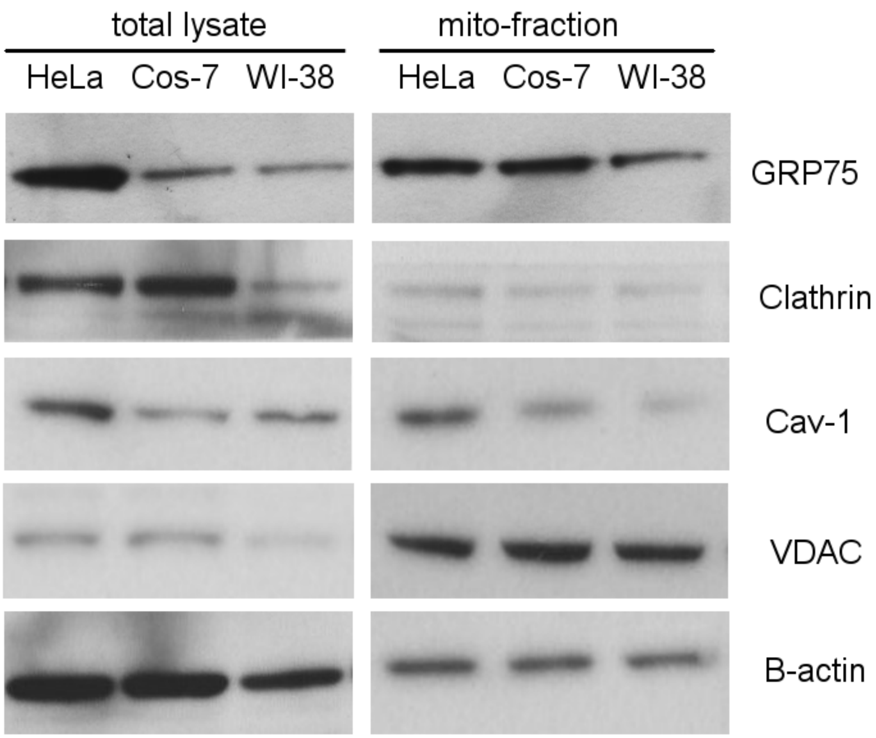

Supplementary Figure 4: Western blot analysis of GRP75 level in cell lines. Subcellular fractions (10ug per lane) extracted from HeLa, Cos-7, and WI-38 cells were blotted with mouse anti-GRP75 Ab, anti-clathrin Ab, anti-caveolin-1, and rabbit anti-VDAC Ab, respectively. mito-: mitochondrial lysate.

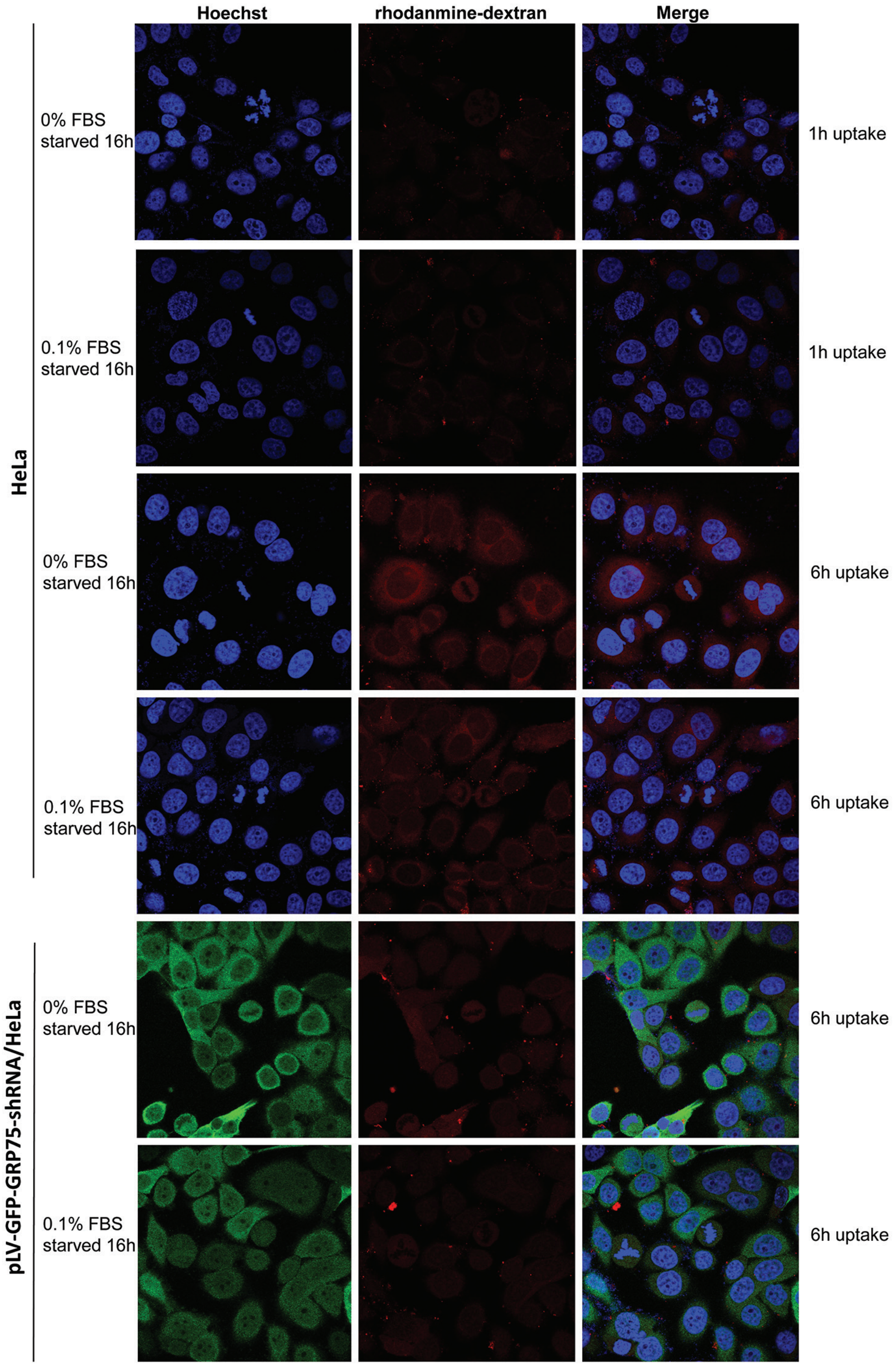

Supplementary Figure 5: Faint macropinocytosis only occurs in heavily starved cells. HeLa and GRP75-shRNA stably transduced HeLa cells were first starved for 16 hours respectively, then incubated with Rhodanmine labeled-dextran (5mg/ml) at 37 °C for different times, rinsed with PBS/0.5M NaCl, and visualized via confocal microscopy. Representative images from three independent experiments are shown. No uptake was detected in non-starved cells (Data not shown).

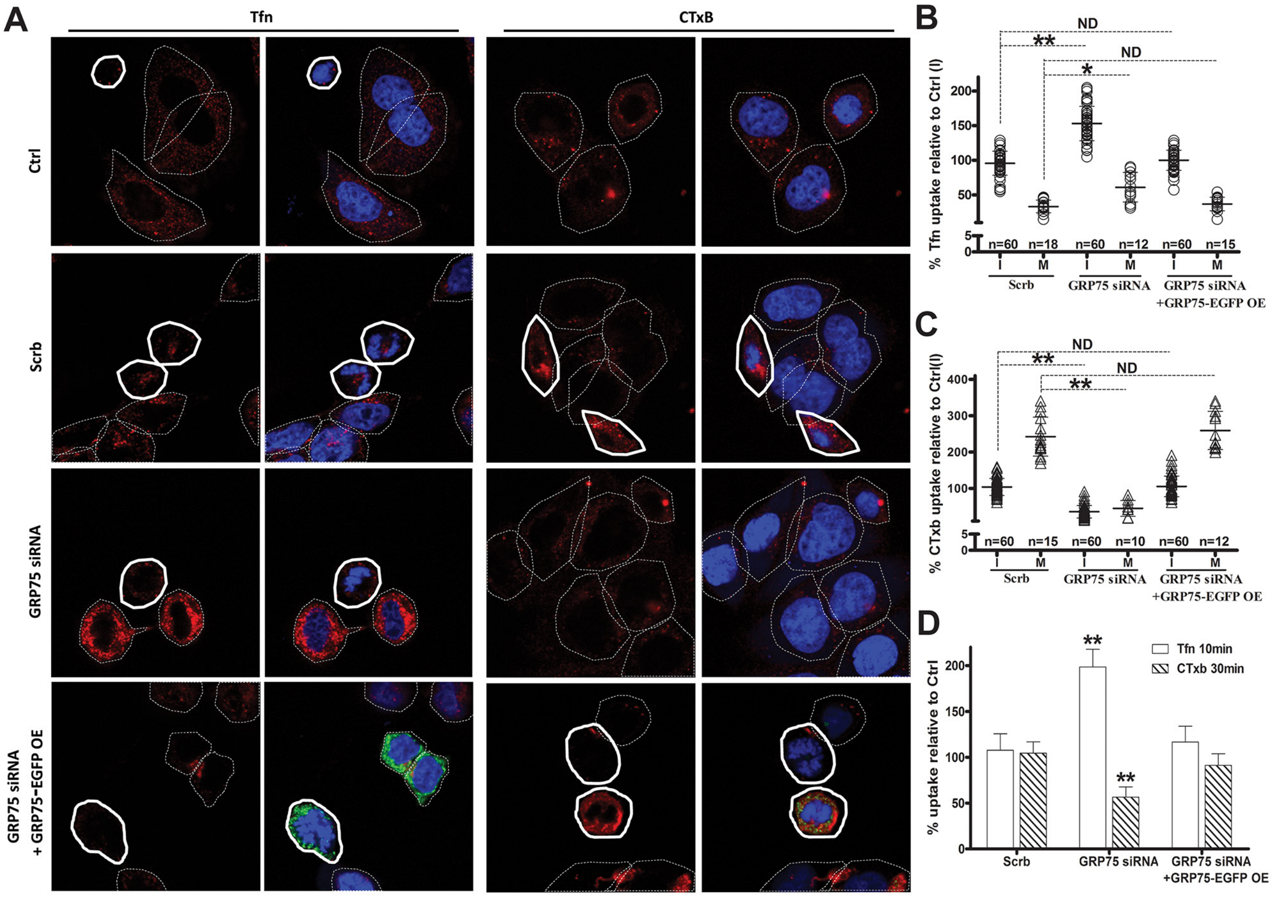

Supplementary Figure 6: GRP75 overexpression reverses the endocytosis changes induced by its knock-down. HeLa cells were transiently transfected with GRP75 siRNA pool, or scramble siRNA (Scrb) as previously described [32], further rescued by GRP75-EGFP plasmid overexpression. The uptake levels of Tfn-AF647 and CTxB-AF647 in cells were determined by confocal imaging analysis, and representative images are shown in (A). Scatterplot depicts the uptake variability of indicated ligands, and the uptake levels in M (polygons with bold solid line) or I phase (polygons with thin dotted line) cells are respectively quantified in (B) and (C) with comparison to that of untransfected cells in I phase as Ctrl (I). At least 60 cells counted for I phase and ≥10 cells counted for M phase for each transfection. (D) The uptake levels of Tfn-AF647 and CTxB-AF647 in tranfected cell population were analyzed and quantified by FACS. 10000 cells counted per sample in each experiment. Statistically significant differences compared with untransfected cells (Ctrl) are shown. ** P <0.01, * P <0.01, ND: no difference.

Supplementary Table 1: Bioinformatics prediction of the subcellular localization of EGFP-fused GRP75 constructs

Constructs	WOLF PSORT	TargetP 1.1			MitoProt II	
	main location of prediction	mTP	SP	other	Loc	Targeting mito probability
EGFP-GRP75	cyto:19,cyto_ nucl:14,nucl:7,mito:2	0.02	0.54	0.76	-	7.39%
GRP75-EGFP	mito:26,cyto_ mito:16.3333,cyto:4.5	0.92	0.01	0.11	M	76.84%
EGFP-ΔS GRP75	cyto:19.5,cyto_ nucl:13.5,nucl:6.5,mito:2	0.02	0.54	0.76	-	7.25%
ΔS GRP75-EGFP	cyto:21,nucl:4,mito:4	0.08	0.14	0.85	-	5.1%

In WOLF PSORT, the bigger the scores, the more the probability; In TargetP, note that the scores are not real probabilities, however, the location with the highest score is the most likely according to TargetP; C, chloroplast; M, mitochondrion; S, secretory pathway; cTP, chloroplast transit peptide; mTP, mitochondrial targeting peptide; SP: secretory pathway signal peptide.

Supplementary Table 2: Cell cycle data of MKT-077 treated cells (n = 3)

Cell-cycle phase distribution (%)		Normal group	DMSO		MKT-077			
			(0.1%)	(0.4%)	(5 uM)	(10 uM)	(20 uM)	(40uM)
HeLa	G1	49.9±0.9	50.6±0.2	53.1±0.2	56.9±0.6	61.1±0.8**	72.6±0.6**	81.2±0.9**
	S	14.5±0.5	20.5±0.7	21.4±0.6	20.0±0.5	21.0±0.2**	14.8±0.6**	6.3±0.6**
	G2/M	33.8±0.6	26.1±0.3	22.7±0.4	21.6±0.9	15.9±0.4**	11.3±0.5**	8.7±0.4**
Cos-7	G1	46.1±0.6	46.4±0.7	48.9±0.8	49.4±0.7	50.7±0.5	53.2±0.1	64.4±0.8
	S	32.5±0.2	33.1±0.3	31.3±0.3	31.8±0.6	33.3±0.8	30.2±0.5	23.6±0.4**
	G2/M	13.8±0.4	13.2±0.4	14.6±0.5	14.1±0.8	12.2±0.2	12.8±0.7	10.4±0.2**

**Significantly different from the normal group ($P < 0.01$).
